# Purification and characterization of β-mannanase from *Aspergillus terreus* and its applicability in depolymerization of mannans and saccharification of lignocellulosic biomass

**DOI:** 10.1007/s13205-016-0454-2

**Published:** 2016-06-18

**Authors:** Hemant Soni, Hemant Kumar Rawat, Brett I. Pletschke, Naveen Kango

**Affiliations:** 1 Enzyme Technology and Molecular Catalysis Laboratory, Department of Applied Microbiology, Dr. Harisingh Gour Vishwavidyalaya (A Central University), Sagar, MP 470003 India; 2 Enzyme Synergy Programme, Department of Biochemistry and Microbiology, Rhodes University, Grahamstown, Eastern Cape 6140 South Africa

**Keywords:** Depolymerization, Lignocellulose, *β*-Mannanase, Oligosaccharides, Saccharification

## Abstract

*Aspergillus terreus* FBCC 1369 was grown in solid-state culture under statistically optimized conditions. β-Mannanase was purified to apparent homogeneity by ultrafiltration, anion exchange and gel filtration chromatography. A purification factor of 10.3-fold was achieved, with the purified enzyme exhibiting specific activity of 53 U/mg protein. The purified β-mannanase was optimally active at pH 7.0 and 70 °C and displayed stability over a broad pH range of 4.0–8.0 and a 30 min half-life at 80 °C. The molecular weight of β-mannanase was calculated as ~49 kDa by SDS-PAGE. The enzyme exhibited *K*_*m*_ and *V*_max_ values of 5.9 mg/ml and 39.42 µmol/ml/min, respectively. β-Mannanase activity was stimulated by β-mercaptoethanol and strongly inhibited by Hg^2+^. The β-Mannanase did not hydrolyze mannobiose and mannotriose, but only mannotetraose liberating mannose and mannotriose. This indicated that at least four mannose residues were required for catalytic activity. Oligosaccharide with a degree of polymerization (DP) three was the predominant product in the case of locust bean gum (16.5 %) and guar gum (15.8 %) hydrolysis. However, the enzyme liberated DP4 oligosaccharide (24 %) exclusively from konjac gum. This property can be exploited in oligosaccharides production with DP 3–4. β-Mannanase hydrolyzed pretreated lignocelluloses and liberated reducing sugars (% theoretical yield) from copra meal (30 %). This property is an important factor for the bioconversion of the biomass.

**Electronic supplementary material:**

The online version of this article (doi:10.1007/s13205-016-0454-2) contains supplementary material, which is available to authorized users.

## Introduction

In plant cell walls, hemicelluloses are the second most abundant carbohydrates after cellulose. Mannans are the second largest group of hemicelluloses after xylan, which appear predominantly in softwoods of gymnosperms and also form a minor component of hardwoods (Puls and Schuseil 1993). These are composed of β-linked mannose sugar-based backbones with variable degrees of side substitutions. These polysaccharides are renewable resources and their enzymatic conversion is of great interest in the field of lignocellulose biotechnology (Soni and Kango 2013). For the majority of bioconversion processes, mannans must be first converted to mannose or manno-oligosaccharides (MOS).

The breakdown of the main chain of mannan is accomplished with the action of β-mannanase (1,4-β-mannan mannohydrolases EC 3.2.1.78), which releases manno-oligosaccharides. Enzymes that actively participate in mannan hydrolysis include β-mannanase (1,4-β-d-mannan mannohydrolase, EC 3.2.1.78), β-mannosidase (1,4-β-d-mannopyranoside hydrolase, EC 3.2.1.25), β-glucosidase (1,4-β-d-glucoside glucohydrolase, EC 3.2.1.21) and α-galactosidase (1,4-α-d–galactoside galactohydrolase, EC 3.2.1.22). Enzymatic synthesis of MOS from low-cost substrate like konjac gum can be developed as a cost-effective method for generating useful nutraceuticals. A bacterial mannanase *MAN5* was used for MOS production form konjac flour and its prebiotic effect has also been described (Al-Ghazzewi et al. 2007). Various reports that reveal the positive effects of MOS on intestinal microflora, intestinal structure and function are available (Baurhoo et al. 2007; Chauhan et al. 2014). Certain MOS possess nutritional values; MOS-based nutrition supplements are widely used in nutrition as a natural additive (Van Zyl et al. 2010). Guar gum derived from seed endosperm of *Cyamopsis tetragonolobus* (guar seed) is very high in viscosity. Enzymatically generated partially hydrolyzed guar gum (PHGG) has low viscosity as compared to guar gum and thus allows its use in enteral products, providing benefits linked with dietary fiber ingestion (Alam et al. 2000; Slavin and Greenberg 2003), which makes the enzyme significant from an industrial point of view. Utilization of lignocellulosic by-products or wastes of agro-industries for the production of biofuel ethanol is very attractive (Chaturvedi and Verma 2013; Gama et al. 2015; Saini et al. 2015). Degradation of agricultural wastes such as wheat bran, wheat straw, copra meal and corn cob requires the homo- and heterosynergistic action of several enzymes. In this study, mannanase was explored for the degradation of lignocellulosic biomass into fermentable monomeric sugars that work as sugar for bioethanol production.

Many microbial β-mannanase production studies have focused on the utilization of pure mannans such as LBG, guar gum and konjac gum as inducers (Puchart et al. 2004; Vijayalaxmi et al. 2013). In addition, low-value mannan-rich substrates such as copra meal, palm kernel cake (PKC), apple pomace, coffee extract and other hemicelluosic biomass like wheat bean and wheat straw can also be used in bioprocesses (Abdeshanian et al. 2010; Kote et al. 2009; Soni et al. 2015).

This current study focused on the formulation of an economical β-mannanase production medium utilizing inexpensive crop straw or agro-industrial wastes that are available in large amounts. Initial pH and moisture content are known to play a crucial role in fungal growth and enzyme production under SSF. In the present study, statistical rotatable central composite design (RCCD) approach of response surface methodology (RSM) was applied to optimized pH and moisture content for the optimized production of mannanase. Attention was then given to the application of β-mannanase for the degradation of mannan for the generation of oligosaccharide, PHGG and saccharification of lignocellulosic biomass.

## Methods

### Materials

Mannobiose (M_2_), mannotriose (M_3_) and mannotetraose (M_4_) standards were purchased from Megazyme (Bray, Ireland). Locust bean gum (LBG), solka floc, glucose, mannose, guar gum, *p*-nitrophenyl-α-d-galactopyranoside, *p*-nitrophenyl-β-d-glucopyranoside, *p*-nitrophenyl-β-d-mannopyranoside, *p*-nitrophenol (pNP) and other chemicals were sourced from Sigma-Aldrich, USA. Copra meal was obtained from Parker Biotech Private Ltd., Tamil Nadu, Chennai, India. Food-grade konjac gum (glucomannan) was obtained from New Foods, Bloomingdale, Illinois, USA. Fenugreek seed (*Trigonella foenum*-*graecum*) meal, *Aloe* *vera* pulp, rice husk, wheat straw and wheat bran were purchased from local markets.

### Microorganism, inoculum preparation and solid-state fermentation

Thermotolerant *A. terreus* FBCC 1369 was isolated from Sagar, MP, India, during a survey of the occurrence of thermophilic fungi from litter and decaying wood (Maijala et al. 2012). It was identified based on the cultural and morphological characteristics. The identity was confirmed using the ITS sequence of the fungus (GenBank: FN811183.1). The strain was maintained on Czapek Dox slants at 4 °C and sub-cultured after every 30 days.

Solid-state fermentation was conducted on mannan-rich low-value particulate substrates, namely, copra meal, fenugreek seed meal, *Aloe vera* pulp, rice husk, wheat straw and wheat bran for the production of β-mannanase. Solid substrate (5 g) was placed in a 250 ml flask and moistened with 5 ml of distilled water. Flasks were then autoclaved at 121 °C for 30 min and inoculated with 1 ml of spore suspension (2 × 10^6^ spores/ml) of *A. terreus* and incubated at 37 °C for 5 days.

Based on the observed β-mannanase activity, copra meal was selected for optimized β-mannanase production by *A. terreus* using one-variable-at-a-time approach. Copra meal (CM) was ground and fractionated using various sieves to collect particle sizes of 2, 1 and 0.5 mm, respectively. The effect of particle size of the substrate was investigated. The effects of glucose, mannose, guar gum, LBG and Solka floc were evaluated as carbon supplements. Urea, yeast extract, peptone and ammonium sulfate were evaluated as nitrogen supplements at a concentration of 1 % (w/v). All the experiments were performed in triplicate and the data represent average ± SD.

### Extraction of β-mannanase and enzyme assays

After incubation, 50 ml citrate buffer (50 mM, pH 5.0) was added to each flask and shaken at 150 rpm for 1 h at 4 °C. For extraction, the entire content of the flask was squeezed through a muslin cloth and the extract was centrifuged at 9000*g* for 15 min at 4 °C. The cell-free clear supernatant was used as a source of mannanase in further studies.

β-Mannanase activity was measured using LBG (0.5 % w/v) as substrate. LBG was dissolved in 50 mM Na-citrate buffer (pH 5.0) by stirring constantly for 1 h at 60 °C. An aliquot of 100 µl enzyme sample was incubated with 900 µl substrate at 50 °C for 10 min. The reaction was stopped by the addition of 1.5 ml dinitrosalicylic acid (DNS) reagent and subsequent boiling for 5 min and reducing sugar was measured at 540 nm against the blank (Miller 1959). One unit of enzyme activity was defined as the amount of enzyme required to produce 1 µmol. of mannose per min under the experimental conditions.

For assay of α-galactosidase, a 900 µl aliquot of *p*-nitrophenyl-α-d-galactopyranoside (2 mM in 50 mM Na-citrate buffer, pH 5.0) was incubated with 100 µl sample at 50 °C for 10 min. The reaction was terminated by adding 0.5 ml of 1 M Na_2_CO_3_ and the absorbance of released *p*-nitrophenol was determined at 400 nm (Maijala et al. 2012). One unit of α-galactosidase was defined as the amount of enzyme that produced 1 µmol *p*-nitrophenol per min. The β-mannosidase and β-glucosidase assay was the same as described for α-galactosidase except that *p*-nitrophenyl-β-d-mannopyranoside (2 mM) and *p*-nitrophenyl-β-d-glucopyranoside (1 mM) were used as substrates, respectively (Maijala et al. 2012).

### Experimental design for the determination of optimum pH and moisture content

Initial pH and moisture content are crucial factors affecting hemicellulases production in solid-state culture (Yin et al. 2013; Sadaf and Khare 2014; Zhang and Sang 2015). Hence, both (A) pH and (B) moisture content were selected to establish the optimum parameters for β-mannanase production by *A. terreus* using an RCCD approach of RSM. The ranges and levels of the variables selected for RSM are listed in Supplementary Table 1. According to RCCD, the total number of experimental combinations is 2*k* + 2*k* + no., where *k* is the number of independent variables and no. is the number of repetitions of the experiments at the center point. A total of 13 sets of experiments, including five center points, were conducted, along with different combinations of two parameters. Each numeric factor was varied over five levels, that is, plus and minus alpha (axial point), plus and minus one (factorial points) and zero (center point).

### Statistical analysis and validation of experimental modeling

The data obtained from RSM was subjected to analysis of variance (ANOVA) for the analysis of the regression coefficient, prediction equations and case statistics. Analysis of data was performed using Design-Expert software (Version 9.0). The experimental results of the RSM were fitted using the second-order polynomial equation:

1 $$Y\, = \,\beta_{0} + \, \sum\limits_{i} {\beta_{i} X_{i} } \, + \, \sum\limits_{ii} {\beta_{ii} X_{i2} } \, + \, \sum\limits_{ij} {\beta_{ij} X_{i} X_{j} } .$$

In this polynomial equation, *Y* is the predicted response, *X*_*i*_ *X*_*j*_ are independent variables, *β*_0_ is the intercept term, *β*_*i*_ is the linear coefficient, *β*_*ii*_ is the quadratic coefficient and *β*_*ij*_ is the interaction coefficient. The statistical model was validated with respect to all variables within the design space. Random sets of two experimental optimized combinations were used to study the β-mannanase production under SSF.

### Purification, protein determination, electrophoresis and zymogram analysis

Crude β-mannanase was concentrated using ultrafiltration employing a 10 kDa cutoff membrane (Millipore). The resulting enzyme preparation was purified by a fast protein liquid chromatography (FPLC) system [AKTA Prime Plus] using a 5 ml Capto^TM^ Q column (Pharmacia Biotech) and eluting with a linear gradient of 0–1 M NaCl in Tris–HCl buffer (pH 8.3) at 1 ml min^−1^. Fractions of β-mannanase activity were pooled, desalted and then concentrated using a Vivaspin 20 ml centrifugal concentrator. The concentrated enzyme was further purified using a Sephacryl™ S-200HR (16/60) column, eluted with 20 mM Tris–HCL buffer (pH 8.3) containing 10 mM NaCl at a flow rate of 0.1 ml min^−1^. Fractions collected were analyzed for β-mannanase activity and analyzed on sodium dodecyl sulfate–polyacrylamide gel electrophoresis (SDS-PAGE). The activity of the purified enzyme was confirmed by zymogram analysis on native PAGE. The protein content was determined by Lowry’s method using bovine serum albumin as standard (Lowry et al. 1950).

SDS-PAGE was performed on a 12 % acrylamide gel (Laemmli 1970) and protein bands were visualized by Coomassie Brilliant Blue G stain. Broad range molecular weight standards (14–175 kDa) were used as size markers. Activity band staining of the purified enzyme was visualized on a 12 % native polyacrylamide gel. After electrophoresis (3–4 h, 70 V), the gel was incubated on a substrate plate (0.5 % LBG in 2 % agar) for 4 h at 50 °C. The zone of β-mannanase activity was visualized using Congo red (0.1 %) stain followed by destaining using aqueous NaCl solution (1 % w/v).

### Characterization of β-mannanase, degradation of mannan polymers, hydrolysis of MOS and saccharification of lignocellulosic substrates

The optimum pH of β-mannanase was determined at 50 °C in 50 mM buffer of varying pH from 4.0 to 10.0. The buffers used were Na-citrate buffer pH (4.0–7.0) and Tris–HCL buffer pH (8.0–10.0). The optimum temperature was determined at pH 7.0 (Na-citrate buffer, 50 mM) in a range of 40–90 °C.

To assay pH stability, β-mannanase was incubated at varying pH (4.0–10.0) for 90 min and the residual β-mannanase activity was determined under standard assay conditions. The thermal stability of β-mannanase was determined after incubation of the enzyme at 50, 60, 70 and 80 °C. Aliquots were withdrawn after 0, 15, 30, 45 and 60 min of incubation, and residual enzyme activity was measured by the standard assay. The effects of various metal ions and inhibitors on β-mannanase activity were determined. Inhibitors such as phenylmethanesulfonyl fluoride (PMSF), ethylene diamine tetraacetic acid (EDTA), 1,10-phenanthroline, mercaptoethanol, SDS and urea at a concentration of 1 mM in 50 mM sodium acetate buffer (pH 5.0) were used. A solution of MgSO_4_, ZnSO_4_, HgCl_2_, MnSO_4_, CaCl_2_, and CuSO_4_ at a concentration of 1 mM was used (Heck et al. 2006). The residual activity in the reaction sample and its corresponding control (i.e., without metal ions or inhibitors) was determined by standard assay.

The substrate specificity of β-mannanase was determined by assaying its activity with guar gum, LBG, konjak gum and copra mannan (0.5 % w/v in 50 mM Na-citrate buffer, pH 5.0) as described earlier. Kinetic parameters were determined using LBG as a substrate in the concentration range of 1–10 mg/ml in 0.05 M Na-citrate buffer (pH 5.0). The Hanse–Woolf plot was drawn for determining the values of *K*_*m*_ and *V*_max_.

The potential of β-mannanase in degrading various mannan polysaccharides was assessed as indicated below. LBG, guar gum and konjak gum (0.5 % w/v in 50 mM Na-citrate buffer, pH 5.0) was incubated with equal volumes (1:1) of substrate and enzyme (25 U/ml) at 50 °C with constant shaking. Samples were withdrawn at intervals of 2, 5, 10 and 20 h and boiled to stop the reaction. Samples were filtered through a membrane filter (pore size 0.45 µm) and the filtrate was analyzed by HPLC (Waters, USA) using a Sugar Pak column, RI detector 2414 and injection valve with capacity of 20 µl (Soni et al. 2015). HPLC-grade water was used in the mobile phase with a flow rate of 0.5 ml/min and a column temperature of 90 °C. The analysis was performed using Empower 2 Build software 2154. β-Mannanase was also employed for mannobiose (M2), mannotriose (M3) and mannotetraose (M4) hydrolysis. A total of 10 units of β-mannanase was incubated with 1 % of the different above-mentioned sugars in 50 mM sodium acetate buffer (pH 5.0) at 50 °C for 24 h. Aliquots were withdrawn after 24 h, boiled for 5 min, and analyzed by thin-layer chromatography (TLC). The reaction mixtures were spotted on a silica gel plate (Merck Silica Gel 60F 254, Germany) and developed twice in a solvent system containing isopropyl alcohol/ethyl acetate/water (2:2:1-v/v/v). Saccharides were detected by heating the plate in an oven after spraying with a mixture of 0.5 % α-naphthol and 5 % sulfuric acid in absolute ethanol (w/v/v) (Kango 2008). Mannose (M1), mannobiose (M2), mannotriose (M3) and mannotetraose (M4) were used as standards.

Lignocellulosic substrates wheat bran (WB), copra meal (CM), wheat straw (WS) and corn cob were saccharified in an incubator shaker at 120 rpm at 50 °C for 24 and 48 h (Maijala et al. 2012). Reducing sugars were determined using the DNS reagent (Miller 1959) in the supernatant after centrifugation. Substrates with suitable particle size (1–2 mm) were also pre-treated with 0.1 N NaOH (alkali treatment) and 0.1 N HCl (20 % w/v) (acid treatment) and autoclaved at 121 °C for 30 min for the observed effect of pre-treatment on saccharification. After pre-treatment, substrates were washed with distilled water until the pH was neutral and dried in an oven. Each substrate was suspended in 50 mM sodium citrate buffer (pH 5.0) and supplemented with a partially purified β-mannanase preparation (20 U/ml); a substrate consistency of 3 % (w/v) was maintained. To avoid loss of sugars due to possible microbial contamination, the reaction mixture also contained 0.02 % NaN_3_. The percentage of saccharification was calculated as indicated below (Baig et al. 2004):

$${\text{Saccharification }} = {\text{sugars}}({\text{mg}}/{\text{ml}}) /{\text{substrate (mg}}/{\text{ml)}} \times {1}00.$$

All the experiments were carried out in triplicate and the results indicate the average of triplicate readings ±SD.

## Results

### Optimization of process parameters

Among the six substrates examined for the production of β-mannanase in SSF, copra meal was observed to be the best supporting 59 U/gds β-mannanase (Supplementary Table 2). Henceforth, copra meal was used for further optimization. Optimized parameters screened through the one-variable-at-a-time approach were as follows: the smallest particle size 0.5 mm of substrate supported a maximum yield of β-mannanase (110 U/gds); addition of 1 % (w/w) pulverized cellulose (solka floc) supported the highest yield of β-mannanase (157 U/gds) comparable to the unsupplemented control (110 U/gds), while among nitrogen supplementation urea supported the highest production of β-mannanase (170.8 U/gds) (Supplementary Fig. 1a–c). The presence of other associated hemicellulases, α-galactosidase (7.2 U/gds) and β-glucosidase (4.3 U/gds) was noted. Such enzyme consortia help to achieve complete depolymerization of mannan. Mannosidase activity was below the detection level.

### Optimization of factors by response surface methodology (RSM)

In this investigation, RSM was applied for the optimization of two crucial factors, viz. pH and moisture for β-mannanase production to study the interaction of these factors at different levels. RSM involving an RCCD was adopted to optimize both parameters for β-mannanase production by *A. terreus*. A set of 13 experiments, including five center points, was carried out. Each numeric factor was varied over five levels (−α, −1, 0, +1, +α). The full experimental plan with respect to their actual and coded forms is listed in supplementary Table 1. The response values (*Y* = β-mannanase activity) in each trial were the average of triplicates. Analysis of variance (ANOVA) was used for analysis of the regression coefficient, prediction equations and case statistics. The experimental results of RSM were fitted using the following second-order polynomial equation 1. In this study, the independent variables were coded as *A* (pH) and *B* (moisture). Thus, the second-order polynomial equation can be represented as follows:

2 $$\beta{\text{-}}{\text{mannanase activity }} = + \, 422.80 - 15.70 \times A + 16.65 \times B - 67.09 \times A^{2} - 128.59 \times B^{2} - 4.25 \times A \times B \times .$$

**Fig. 1:**
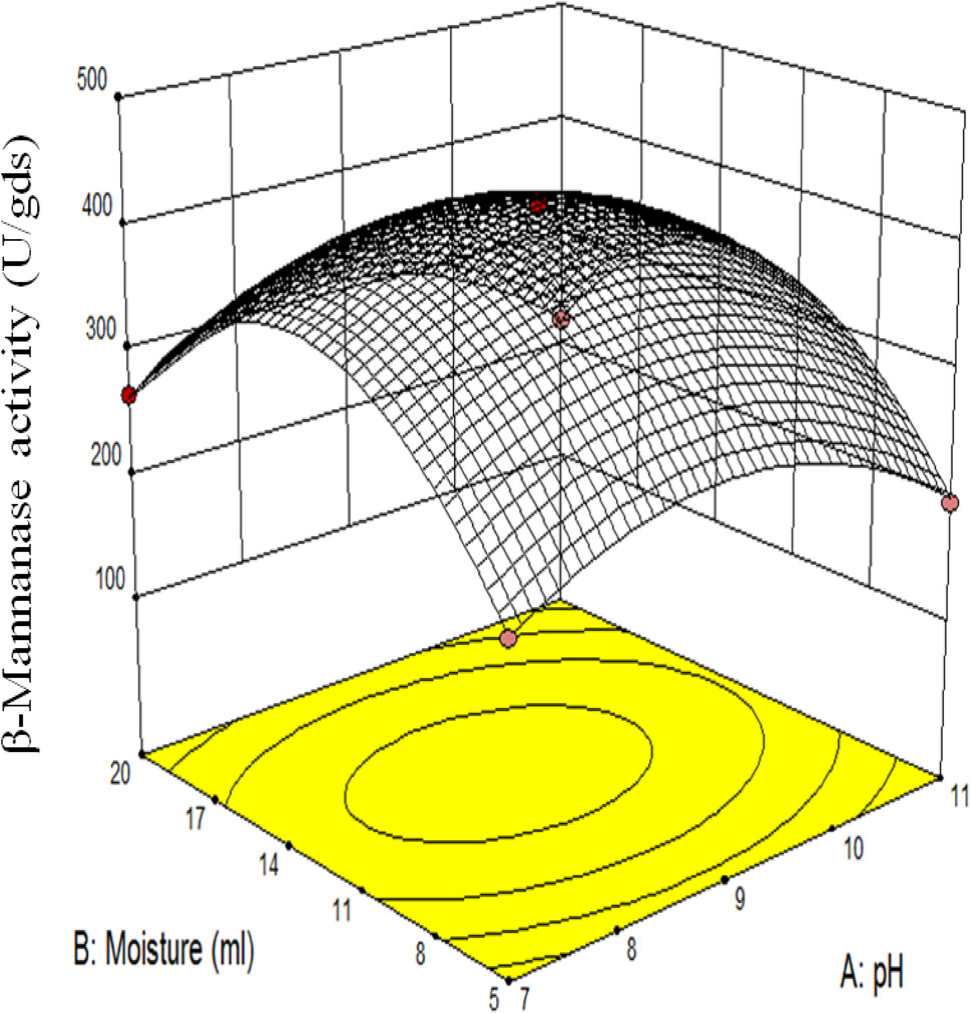
3D response surface plot for production of β-mannanase as a function of ***A*** pH and ***B*** moisture content

The statistical significance of the second-order polynomial equation (Eq. 2) was checked by Fisher distribution (*F* test) (ANOVA) and the results are shown in Supplementary Table 3. The “predicted *R*-squared” value of 0.997 is in reasonable agreement with the “adjusted *R*-squared” value of 0.99. This indicated a good agreement between the observed and predicted values. In this case, *A*, *B*, *AB*, *A* ^2^ and *B* ^2^ were significant model terms. Moreover, “lack of fit (LOF) *F* value” of 0.08 implies that it was not significant relative to the pure error. Non-significant LOF indicated a good fitness of model. Predicted vs. actual plot (Supplementary Fig. 2) represents a high degree of similarity that was observed between the predicted and experimental values. The 3D response surface curve and its respective 2D contour plot (Fig. 1) illustrate the interaction between both factors and indicate the optimum value of each factor for maximum response in terms of β-mannanase production yield (U/gds). The plot was obtained from the pairwise combination of independent factors. Increasing the moisture of the medium from 1.8 to 12.5 ml significantly increased the β-mannanase production yield from 145 to 423 U/gds, but thereafter no significant increases in β-mannanase production yield was observed. It was also observed that when the pH was increased beyond level “0”, the β-mannanase production yield decreased (Std. run 3, 4 and 6 in Supplementary Table 1).

### Characterization of β-mannanase

**Fig. 2:**
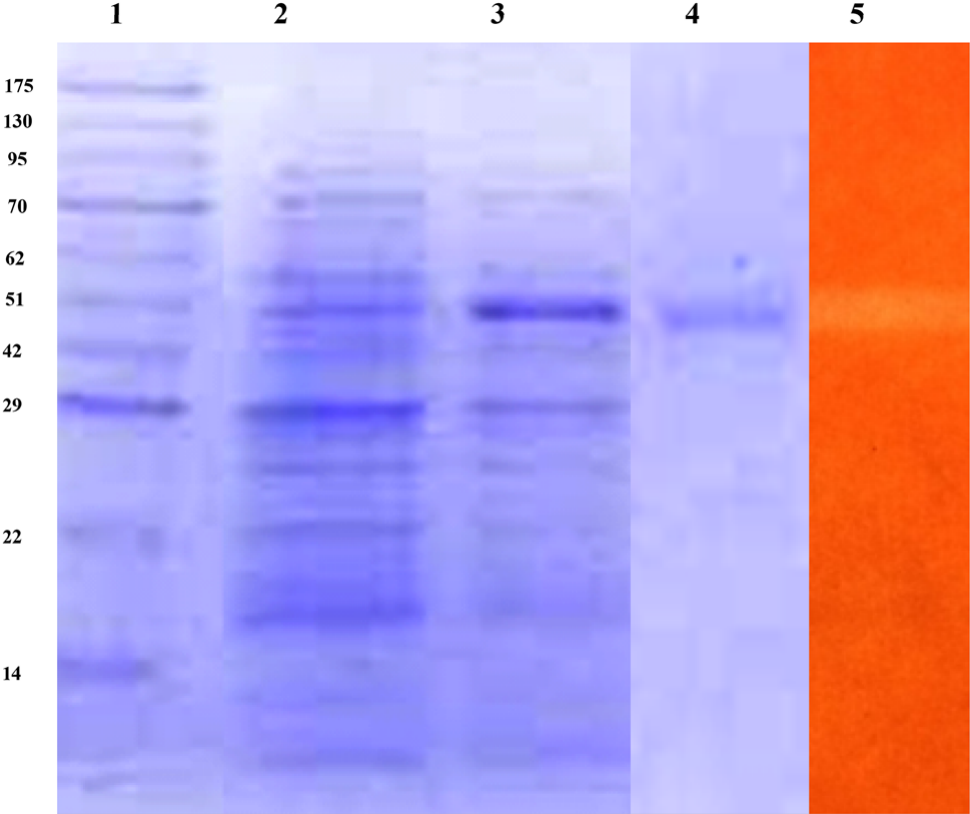
SDS-PAGE and native PAGE (zymogram) analysis of purified β-mannanase from *A. terreus*; *lane* 1 standard protein marker; *lane* 2 crude protein, *lane* 3 protein after ion exchange chromatography; *lane* 4 purified β-mannanase; and *lane* 5 zymogram of the β-mannanase purified from *A. terreus*

In the present study, β-mannanase extracted from a solid-state culture of *A. terreus* was purified by ultrafiltration, anion exchange and gel filtration. The results of the purification steps are summarized in Table 1. Purified β-mannanase had a specific activity of 53.75 U/mg. This preparation was further used for characterization of β-mannanase. SDS-PAGE of the purified protein revealed a single band suggesting that β-mannanase from *A. terreus* FBCC 1369 is a monomeric polypeptide with an estimated molecular weight of ~49 kDa. This was further confirmed by hydrolysis on gel containing mannan substrate through zymogram (Fig. 2).

**Table 1:** Summary of β-mannanase purification from *A. terreus* FBCC 1369

Purification step	Total activity (U)	Specific activity (U/mg)	Yield (%)	Purification (fold)
Culture filtrate	2115	5.2	100	1
Ultrafiltration	765	26.3	36	5.05
Ion exchange	360	30.6	17	5.8
Gel filtration	223	53.75	10	10.3

**Fig. 3:**
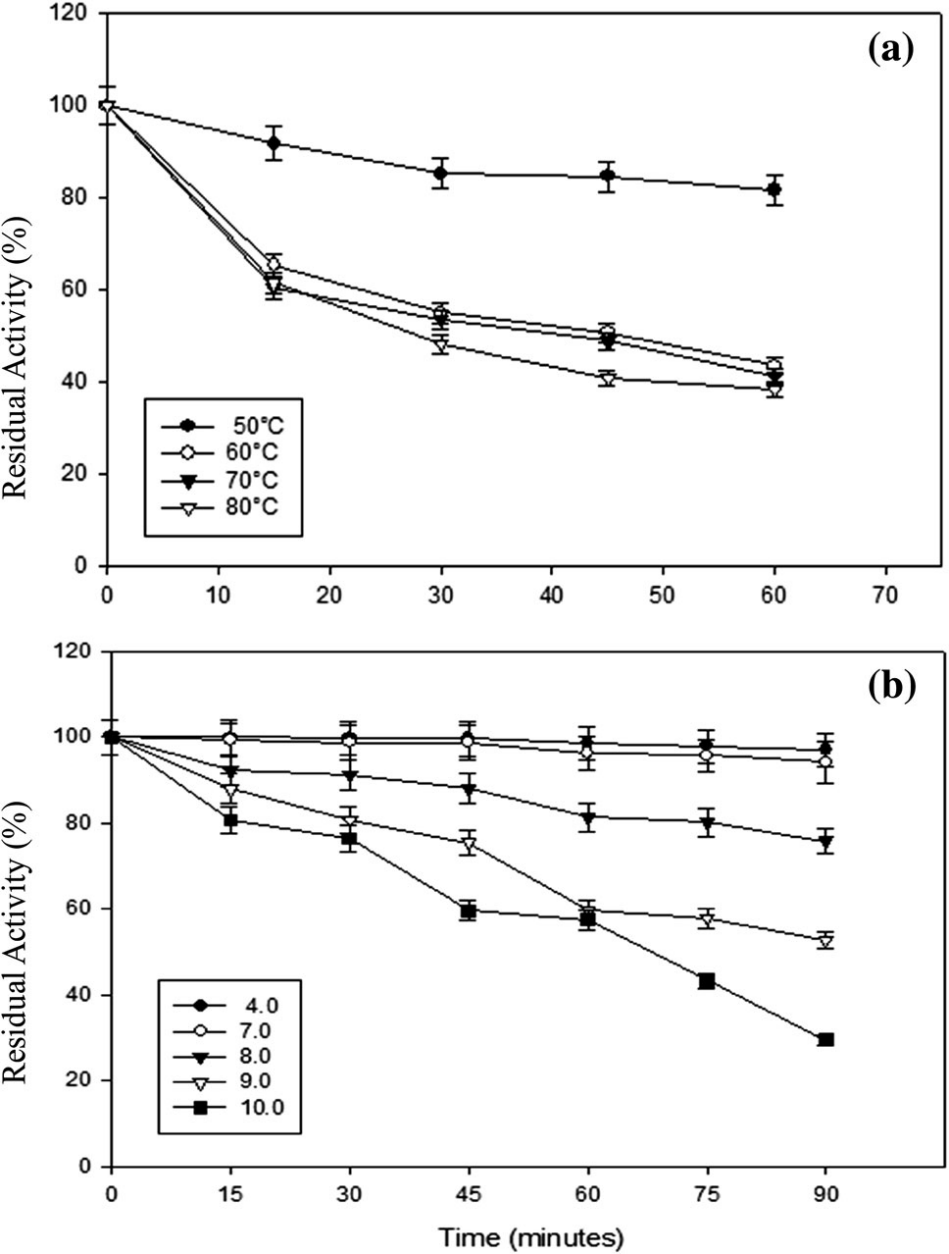
Temperature (**a**) and pH (**b**) stability of *A. terreus* FBCC 1369 β-mannanase. Data points indicate the means of triplicate values ± SD

For industrial use, pH and temperature are the important factors which could affect the catalytic efficiency of an enzyme. Optimum pH and temperature values for β-mannanase activity were pH 7.0 and 70 °C, respectively (Supplementary Fig. 3a, b). The stability profile of β-mannanase indicated this to be a relatively thermostable enzyme active over a wide pH range. β-Mannanase was stable up to 1 h at 50 °C, retaining 85 % activity. The half-life of this enzyme at 80 °C was about 30 min (Fig. 3a). The enzyme was stable in the pH range of 4.0–7.0 (Fig. 3b) retaining full activity after 90 min incubation. The enzyme retained more than 50 % activity at pH 10.0 after 60 min of incubation.

Among various substrates, *A. terreus* β-mannanase hydrolyzed LBG most efficiently, suggesting that LBG is the most suitable substrate for its action (Supplementary Fig. 4). *K*_*m*_ and *V*_max_ values were observed to be 5.9 ± 0.3 mg/ml and 39.42 ± 0.4 µmol/ml/min, respectively, on LBG.

The effect of potential inhibitors or activators on purified β-mannanase is shown in Supplementary Table 4. Results showed that Hg^2+^, Zn^2+^ PMSF and EDTA were strong inhibitors of enzyme activity, while Ca^2+^, urea and KCl had a slight inhibitory effect on its activity. 1,10-Phenanthroline, Cu^2+^ and Mg^2+^ did not affect the enzyme activity while β-mercaptoethanol, a reducing reagent, enhanced enzyme activity by 30 %.

### Validation of the model

An attempt was made to maximize the productivity of β-mannanase while keeping the moisture and pH ‘in the range’. Using these criteria, a solution pH 8.8 and moisture 13 ml with maximum response were selected and experiments were conducted. The observed response (422 ± 1.8 U/gds yield of β-mannanase) was near the predicted outcome (423 U/gds yield of β-mannanase). The production of enzyme predicted by the quadratic model equation, and that recorded experimentally, were in good agreement, and thus the model was valid. The production attained after statistical optimization was sevenfold higher than that attained under unoptimized conditions (Supplementary Table 5).

### Degradation of mannan polymers and hydrolysis of MOS

**Fig. 4:**
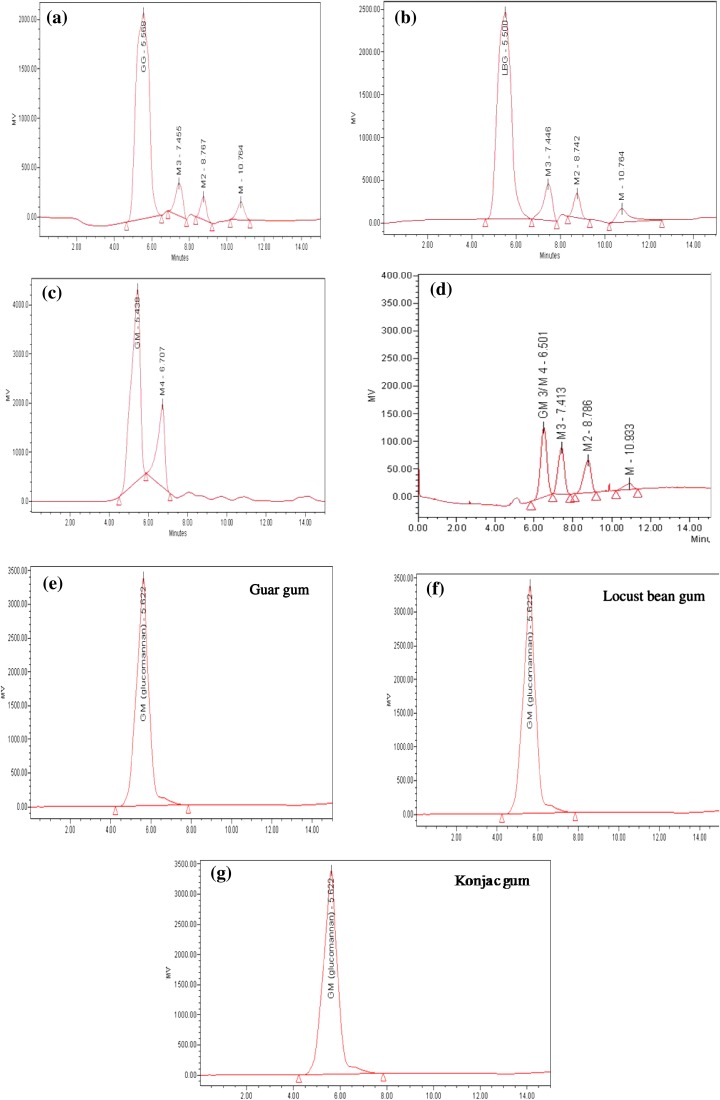
HPLC analysis of mannan hydrolysis: equal volume of enzyme and substrate (1:1) were incubated at 50 °C under constant shaking. **a** Hydrolysis of guar gum (GG); **b** hydrolysis of LBG; **c** hydrolysis of konjac gum; **d** standards, mannose (M), mannobiose (M2), mannotriose (M3), mannotetraose (M4), HPLC analysis of substrates; **e** guar gum; **f** locust bean gum; **g** konjac gum

**Fig. 5:**
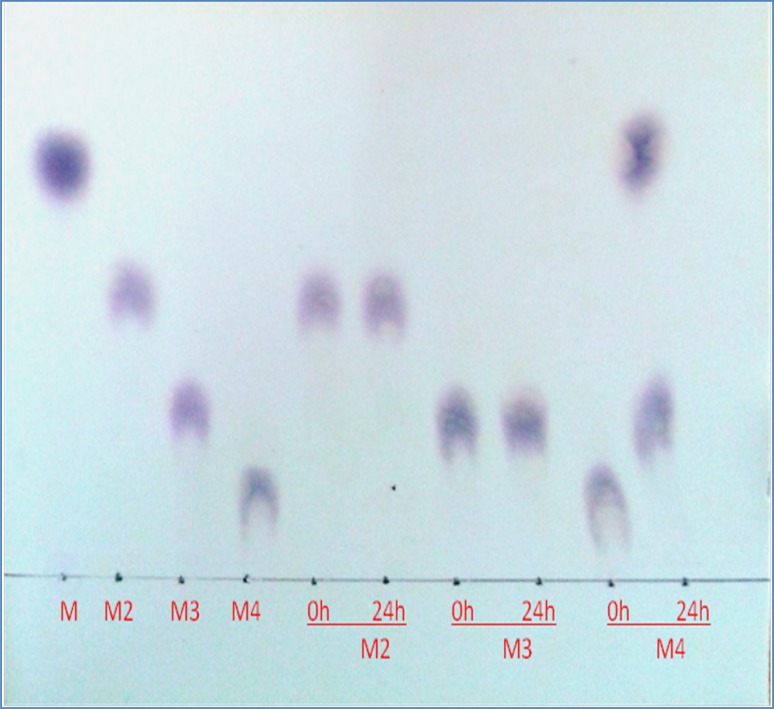
TLC of end product analysis of action of purified β-mannanase on M2, M3 and M4 (10 U/ml β-mannanase was incubated with 1 % (M2) mannobiose (M3) mannotriose and mannotetraose (M4) at 50 °C in 50 mM sodium acetate buffer (pH 5) for 24 h. M, M2, M3 and M4 were used as standards of MOS (1 % w/v in sodium acetate buffer, pH 5)

The degradation of various mannan polymers such as LBG, guar gum and konjac powder by the preparation of mannanase of *A. terreus* was performed. As shown in Fig. 4a–c, mannanase preparation was able to degrade different types of mannan polymers, such as 32, 28 and 24 % LBG, guar gum (galactomannans) and konjac gum (glucomannan), respectively. The degradation of LBG and guar gum resulted in the formation of a mixture of mannose (M), mannobiose (M2) and oligosaccharide with the degree of polymerization (DP) of the three sugars, while hydrolysis of konjac gum yielded predominantly oligosaccharide with DP of four as the main product (Table 2). Purified β-mannanase was incubated with different MOS and the products were resolved by TLC. TLC analysis confirmed that β-mannanase cannot cleave the glycosidic linkage in M2 and M3 even after a prolonged incubation period of 24 h; however, it efficiently hydrolyzed mannotetraose forming M3 and M (Fig. 5). The results indicated that β-mannanase was highly endo-acting and required at least four mannose residues for hydrolytic activity.

**Table 2:** End product hydrolysis profile of different types of mannan by *A. terreus* FBCC 1369 β-mannanase

Substrate	M	M2	MOS (DP 3)	MOS (DP 4)
Locust bean gum powder (galactomannan)	4.9 %	10.8 %	16.5 %	–
Guar gum powder (galactomannan)	5.9 %	6.8 %	15.8 %	–
Konjac gum powder (glucomannan)	–	–	–	24 %

*M* mannose, *M2* mannobiose, *MOS* manno-oligosaccharides, *DP* degree of polymerization

### Saccharification of lignocellulosics

Preparation of mannanase from *A*. *terreus* potentially saccharified wheat bran, followed by copra meal, wheat straw and corn cob. It can be observed that the saccharification of alkali-pretreated substrates yielded more reducing sugar than untreated substrates. The results are summarized in Table 3. It indicated that pretreatment of substrates could result in improvement of reducing sugar yield.

**Table 3:** Saccharification of lignocellulosic substrates using a mannanase preparation from *A. terreus* FBCC 1369

Substrate	Treatment	Reducing sugars (%) liberated after reaction times	
		24 h	48 h
Wheat bran	NW	5.5	7.9
	AcT	10.4	15
	AlT	32.2	37.5
Copra meal	NW	5.5	5.0
	AcT	1.5	13
	AlT	25.3	30.0
Wheat straw	NW	3.0	5.5
	AcT	3	5.5
	AlT	15	17.8
Corn cob	NW	2.3	2.4
	AcT	2.8	3.3
	AlT	3.4	3.7

*NW* normal washing, *AcT* acidic treatment, *AlT* alkali treatment

## Discussion

Thermotolerant fungus *A. terreus* FBCC 1369 produced 59 U/gds titer of β-mannanase under non-optimized conditions in solid-state culture on copra meal. Optimization of various parameters, viz. the particle size of substrate and carbon and nitrogen supplementation, was carried out. The smallest particle size of 0.5 mm supported the maximum β-mannanase production and is similar to the findings of a particle size of 0.6 mm being suitable for xylanase production by *Sporotrichum thermophile* (Sadaf and Khare 2014). Among the carbon supplements examined, pulverized cellulose (solka floc) supported higher yields of β-mannanase. Similar levels of induction were also observed in *Myceliophthora fergusii* MTCC 9293 (Maijala et al. 2012). Glucose and mannose supplementation clearly repressed β-mannanase production. Supplementation of complex galactomannans like guar gum and LBG also lowered the enzyme yield significantly.

*Aspergillus terreus* produced a higher yield of β-mannanase at alkaline pH (9.0), with obvious luxuriant growth in SSF, indicating its alkaliphilic nature.

High titers of β-mannanase 422 U/gds were attained after statistical optimization and the results suggest that copra meal as solid can be used alone without any supplementation for the efficient production of β-mannanase.

Purified β-mannanase was a ~49 kDa monomeric protein (Fig. 2). β-Mannanases of molecular mass in the range of 40–60 kDa are usually monomeric in nature (Ademark et al. 1998; Luo et al. 2009; Lim et al. 2012). Most of the fungal β-mannanases characterized so far exhibited optimal activity in the acidic pH range of 4.0–5.0 (Christgau et al. 1994; Benech et al. 2007; Lim et al. 2012; Katrolia et al. 2013), while β-mannanase from *A. terreus* FBCC 1369 was maximally active at pH 7.0. In the present study, the optimum temperature for β-mannanase was 70 °C, which is similar to that reported for β-mannanase of *Aspergillus aculeatus* (60–70 °C) (Regalado et al. 2000) and higher than those of other Aspergilli viz. *Aspergillus* *niger* (60 °C), *Aspergillus* *sulphureus* (50 °C) and the thermotolerant *Aspergillus fumigatus* (60 °C) (Regalado et al. 2000; Puchart et al. 2004; Chen et al. 2007). In this study, the half-life of β-mannanase at 80 °C was about 30 min, while β-mannanase MANI and MANII from the thermotolerant *Aspergillus fumigatus* were rapidly inactivated above 60 °C (Puchart et al. 2004). The properties of some fungi β-mannanases, in comparison to that of the strain used in the present study, are summarized in Supplementary Table 6.

Ions that react with sulfydryl groups such as Hg^2+^ generally inhibit enzyme activity. In this study, inhibition of β-mannanase revealed that there was an important cysteine residue in or near the active site of the enzyme (Chevero et al. 2001). As was the case in this study, enhancement of enzyme activity by β-mercaptoethanol has also recently been reported for some enzymes (Sharma and Satyanarayana 2013).

Mannanase displayed a great deal of variation in their ability to degrade a diverse range of mannans from different sources. Based on the amounts of hydrolysis products of different substrates, the hydrolysis degree of mannanase against various substrates was in the order of LBG (32 %) > guar gum (28 %) > konjac powder (24 %). The results showed that mannanase was more active in degrading galactomannan than glucomannan. The results also suggested that β-mannanase possesses high endo-β-mannanase activity and releases oligosaccharides with DP of 3–4. It has been reported that oligosaccharides from hydrolysis of mannans can act as prebiotics for positive effects on the growth of chickens and prevention of infection. MOS are able to interfere in bacterial attachment (of *Salmonella* and *E. coli*). In the intestinal tract, however, these oligosaccharides selectively promote the growth of beneficial bacteria, especially *Lactobacillus* and *Bifidobacterium* (Chauhan et al. 2014; Dhawan et al. 2015). Thus, the production of oligosaccharides with DP 3–4 makes *A. terreus* β-mannanase a good candidate for potential application in the feed industry.

The results of substrate specificity studies indicated that *A. terreus* β-mannanase exhibited high activity toward LBG (defined as 100 %) followed by guar gum (86 %), konjac gum (52 %) and copra mannan (8 %) (Supplementary Fig. 4). This is in strong contrast to the β-mannanase from *Bacillus circulans* NT 6.7 (Piwapankaew et al. 2014), which displayed activity only on LBG, with no activity on guar gum. β-Mannanase from *Reinkea* sp. KIT-Y010 did not display any activity on either LBG or guar gum, but was able to hydrolyze linear mannans (konjac gum) much more efficiently (Hakamada et al. 2014).

HPLC results showed that the hydrolysis of LBG was approximately twofold higher compared to *A*. *awamori* K4 β-mannanase (Kurakake and Komaki 2001). Guar gum hydrolysis indicated that this β-mannanase could be useful in the preparation of partially hydrolyzed guar gum (PHGG), a clinical nutrition supplement useful in the treatment of irritable bowel syndrome (IBS) and in the manufacture of enteral products and beverages (Alam et al. 2000; Slavin and Greenberg 2003).Exclusive generation of DP4 manno-oligosaccharide from konjac gum, with negligible mannose, suggests the potential of* A. terreus* β-mannanase, in industrial prebiotic preparations. Zhang et al. (2009) have reported the formation of oligosaccharides (DP 2–6), with negligible mannose, from konjac flour using bacterial β-mannanase.

Saccharification of lignocellulosic biomass using partially purified β-mannanase showed that alkali pre-treatment enhanced saccharification of substrates as compared to untreated substrates. It indicated that pretreatment could result in increased degradation of lignocellulosic materials. Similar results were reported by Zhang and Sang (2015) and Liao et al. (2015) using xylanase. Recently, Cameron et al. (2015) used crude mannanase from *Penicillium* sp. and observed improved saccharification of *Pinus radiate*.

## Conclusion

The high β-mannanase yield on low-value copra meal, exclusive generation of DP 4 oligosaccharide from konjac gum, formation of partially hydrolyzed guar gum (PHGG) and a 30 min half-life at 80 °C make *A. terreus* β-mannanase an attractive enzyme for the nutraceutical, food and paper industries. Copra meal is rich in indigestible mannan, cannot be used directly as animal feed (poultry and pigs) and its disposal causes pollution. In the present study it is utilized as substrate for the production of mannanase. The residues left after SSF have reduced galactomannan content and can be used as feed for monogastric animals. This study provides a suitable valorization solution for the utilization and management of copra-oil industry waste which causes pollution.

## Electronic supplementary material

Below is the link to the electronic supplementary material.

Supplementary material 1 (DOCX 118 kb)
