# Supplementary material for: Purification and characterization of β-mannanase from Aspergillus terreus and its applicability in depolymerization of mannans and saccharification of lignocellulosic biomass
Source: 3 Biotech. 2016 Jun 18;6(2):136. doi: 10.1007/s13205-016-0454-2 (PMC4912962; doi:10.1007/s13205-016-0454-2)
Supplement: Supplementary file 1 — Supplementary material 1 (DOCX 118 kb) [file 13205_2016_454_MOESM1_ESM.docx]

**Appendix A**. Supplementary data

**Purification and characterization of β-mannanase from *Aspergillus terreus* and its applicability in depolymerization of mannans and saccharification of lignocellulosic biomass**

**Hemant Soni^1^,** **Hemant Kumar Rawat^1^, Brett I. Pletschke^2^ and Naveen Kango^1^***

^1^Enzyme Technology and Molecular Catalysis Laboratory, Department of Applied Microbiology, Dr. Harisingh Gour Vishwavidyalaya (A Central University)

Sagar (M.P.) 470003

^2^Enzyme Synergy Programme, Department of Biochemistry and Microbiology, Rhodes University, Grahamstown 6140, Eastern Cape, South Africa

* Corresponding author

nkango@gmail.com

Contact num +919425635736

**Figure 1** Effect of various process parameters on β-mannanase production by *A. terreus* FBCC 1369 on copra meal in SSF. The incubation time and temperature was 5 days and 37°C, respectively a) Effect of particle size of copra meal b) Effect of carbon supplementation (1% w/v) with 0.5 mm particle size (KK: konjac gum, GG: guar gum, SF: solka floc, LBG: locust bean gum) c) Effect of nitrogen supplementation (1% w/v) with 0.5 mm particle size (YE: yeast extract, AS: ammonium sulphate)

**Figure 2** Predicted vs. actual response plot of the quadratic model used for β-mannanase production yield (U/gds)

**Figure 3** pH (a) and temperature (b) optima of *A. terreus* FBCC 1369 endo-β-mannanase. Data points indicate the means of triplicate values ± SD.

**Figure 4** Substrate specificity of *A. terreus* FBCC 1369 mannanase; 0.5% w/v of different mannans was incubated with 20 U of β-mannanase at 50°C for 10 min. Data points indicate the means of triplicate values ± SD.

**Figure-1**

**Figure-2**

**Figure-3**

**Figure-4**

Table 1.

C

Process variables used in the RCCD approach of RSM, showing the levels of variables and prediction and actual β-mannanase production. Data points indicate the means of triplicate values ± SD.

C

| Std. Run | Level | A:pH | B Moisture(ml) | β-mannanase production  (U/gds) | |
| --- | --- | --- | --- | --- | --- |
|  | -α | 6.2 | 1.9 | Predicted | Actual |
|  | -1 | 7 | 10 |  |  |
|  | 0 | 9 | 12.5 |  |  |
|  | +1 | 11.8 | 15 |  |  |
|  | +α | 84.14 | 23 |  |  |
| 1 |  | 7 | 5 | 221.87 | 221±4.3 |
| 2 |  | 11 | 5 | 199.35 | 195±4.7 |
| 3 |  | 7 | 20 | 263.79 | 265±5.3 |
| 4 |  | 11 | 20 | 223.77 | 222±5.8 |
| 5 |  | 6.2 | 12.5 | 310.8 | 310±7.1 |
| 6 |  | 11.8 | 12.5 | 266.58 | 270±3.3 |
| 7 |  | 9 | 1.9 | 142.24 | 145±2.9 |
| 8 |  | 9 | 23. | 189.15 | 189±1.8 |
| 9 |  | 9 | 12.5 | 422.8 | 420±6.3 |
| 10 |  | 9 | 12.5 | 422.8 | 423±6.7 |
| 11 |  | 9 | 12.5 | 422.8 | 423±6.1 |
| 12 |  | 9 | 12.5 | 422.8 | 425±5.9 |
| 13 |  | 9 | 12.5 | 422.8 | 423±5.7 |

Table 2.

Production of mannan degrading enzymes on various low value substrates. Data points indicate the mean of triplicate values ± SD.

| Substrate | β-Mannanase  U/gds | α-Galactosidase  U/gds | β-Glucosidase  U/gds |
| --- | --- | --- | --- |
| CM | 59 ± 2.3 | 7 ± 1.1 | 4 ± 1.5 |
| Wheat bran | 5.1 ± 0.5 | 4.4 ± 1.1 | 5.2 ± 1.4 |
| Fenugreek seed powder | 25.2 ± 3.4 | 11 ± 1.5 | 12 ± 1.8 |
| Rice husk | 0.5 ± 0.3 | 0.7 ± 0.1 | 0.3 ± 0.1 |
| Wheat straw | 2.9 ± 1.2 | 1.2 ± 0.2 | 1.3 ± 0.2 |
| Aloe vera pulp | 0.3 ± 0.1 | BD | BD |

BD: below detection level, CM: copra meal

Table 3.

Analysis variance (ANOVA) of the quadratic model of RCCD for β-mannanase production.

| Source | Sum  of Squares | DF | Mean Square | F Value | p-value (Prob > F) | | |
| --- | --- | --- | --- | --- | --- | --- | --- |
| Model | 1.372E+005 | 5 | 27440.44 | 3377.77 | < 0.0001 | significant | |
| A-pH | 1970.93 | 1 | 1970.93 | 242.61 | < 0.0001 |  | |
| B-Moisture | 2218.63 | 1 | 2218.63 | 273.10 | < 0.0001 |  | |
| AB | 72.25 | 1 | 72.25 | 8.89 | 0.0205 |  | |
| A^2^ | 31309.44 | 1 | 31309.44 | 3854.02 | < 0.0001 |  | |
| B^2^ | 1.150E+005 | 1 | 1.150E+005 | 14158.87 | < 0.0001 |  |  |
| Residual | 56.87 | 7 | 8.12 | 3377.77 |  |  | |
| Lack of Fit | 44.07 | 3 | 14.69 |  |  |  | |
| Pure Error | 12.80 | 4 | 3.20 | 4.59 | 0.0875 | not significant | |
| Cor Total | 1.373E+005 | 12 | 27440.44 |  |  |  | |
| Model | 1.372E+005 | 5 |  |  |  |  | |

R^2^= 0.9996; Pred R^2^= 0.997; Adj R^2^= 0.9993; CV%= 0.94

Table 4.

Effect of inhibitors and metal ions at (1 mM concentration)on *A. terreus* FBCC 1369 β-1,4-mannanase activity.

Data points indicate the mean of triplicate values ± SD.

| Inhibitors/metal ions | Residual activity (%) |
| --- | --- |
| Control | 100 ± 0.4 |
| 1,10-Phenanthroline | 107 ± 0.3 |
| PMSF | 58 ± 0.5 |
| EDTA | 66 ± 0.2 |
| Mercaptoethanol | 130 ± 0.1 |
| SDS | 98 ± 0.5 |
| Urea | 94 ± 0.5 |
| KCl | 96 ± 0.3 |
| Mg^2+^ | 111 ± 0.5 |
| Hg^2+^ | 42 ± 0.1 |
| Zn^2+^ | 49 ± 0.3 |
| Cu^2+^ | 101 ± 0.4 |
| Ca^2+^ | 93 ± 0.4 |
| Mn^2+^ | 111 ± 0.3 |

| **Approaches** | **Mannanase production (U/gds)** | **Fold increase** |
| --- | --- | --- |
| Unoptimized | 57 ± 2.3 | 1.0 |
| One variable at time-  approach | 170 ± 2.8 | 2.8 |
| Statistical approach | 422 ± 1.8 | 7.2 |

Table 5.

Analysis of β-mannanase production by *A. terreus* FBCC 1369 using CM as substrate. Data points indicate the mean of triplicate values ± SD.

| Organism | Optimum temperature  and pH  for activity | | Thermostability | pH stability | References |
| --- | --- | --- | --- | --- | --- |
|  | (°C) | pH |  |  |  |
| *Aspergillus awamori* K4 | 80 | 5.0 | NR | NR | Kurakake and Komaki, 2001 |
| *Penicillium oxalicum SO* | 60 | 5.0 | NR | NR | Kurakake et al., 2006 |
| *Aspergillus fumigatus*  (MANI, MANII) | 60 | 4.5 | 55°C/300min  and inactive >60 °C | 4.5-8.5 | Puchart et al., 2004 |
| *Aspergillus sulphureus* | 50 | 2.4 | 50%/40°C/360 min | NR | Chen et al., 2007 |
| *Aspergillus oryzae* NRRL | 55 | 5.5 | 100%/55°C/15 min | 100%/pH4-6/120 min/45 °C | Fattah et al., 2009 |
| *Penicillium occitanis* pol6 | 40 | 4.0 | >80%/50 °C/30 min | >70%/pH 4-10/ 24h | Blibech et al. 2011 |
| *A. terreus* FBCC1369 | 70 | 7.0 | >80%/50°C/60 min and 40%/80 °C/60min | >80%/pH4-8/ 90min and>50% /pH9-10/60min | Present study |

Table 6.

Comparison of *A. terreus* FBCC 1369 mannanase temperature and pH optima and stabilities to that of other fungal β-mannanases
